# Advances in the application of Raman spectroscopy in haematological tumours

**DOI:** 10.3389/fbioe.2022.1103785

**Published:** 2023-01-10

**Authors:** Haoyue Liang, Ruxue Shi, Haoyu Wang, Yuan Zhou

**Affiliations:** ^1^ State Key Laboratory of Experimental Hematology, National Clinical Research Center for Blood Diseases, Haihe Laboratory of Cell Ecosystem, Institute of Hematology and Blood Diseases Hospital, Chinese Academy of Medical Sciences and Peking Union Medical College, Tianjin, China; ^2^ Tianjin University of Traditional Chinese Medicine, Tianjin, China

**Keywords:** Raman spectroscopy, haematological tumours, leukaemia, lymphoma, myelodysplastic syndromes, multiple myeloma

## Abstract

Hematologic malignancies are a diverse collection of cancers that affect the blood, bone marrow, and organs. They have a very unpredictable prognosis and recur after treatment. Leukemia, lymphoma, and myeloma are the most prevalent symptoms. Despite advancements in chemotherapy and supportive care, the incidence rate and mortality of patients with hematological malignancies remain high. Additionally, there are issues with the clinical diagnosis because several hematological malignancies lack defined, systematic diagnostic criteria. This work provided an overview of the fundamentals, benefits, and limitations of Raman spectroscopy and its use in hematological cancers. The alterations of trace substances can be recognized using Raman spectroscopy. High sensitivity, non-destructive, quick, real-time, and other attributes define it. Clinicians must promptly identify disorders and keep track of analytes in biological fluids. For instance, surface-enhanced Raman spectroscopy is employed in diagnosing gene mutations in myelodysplastic syndromes due to its high sensitivity and multiple detection benefits. Serum indicators for multiple myeloma have been routinely used for detection. The simultaneous observation of DNA strand modifications and the production of new molecular bonds by tip-enhanced Raman spectroscopy is of tremendous significance for diagnosing lymphoma and multiple myeloma with unidentified diagnostic criteria.

## Introduction

**FIGURE 1:**
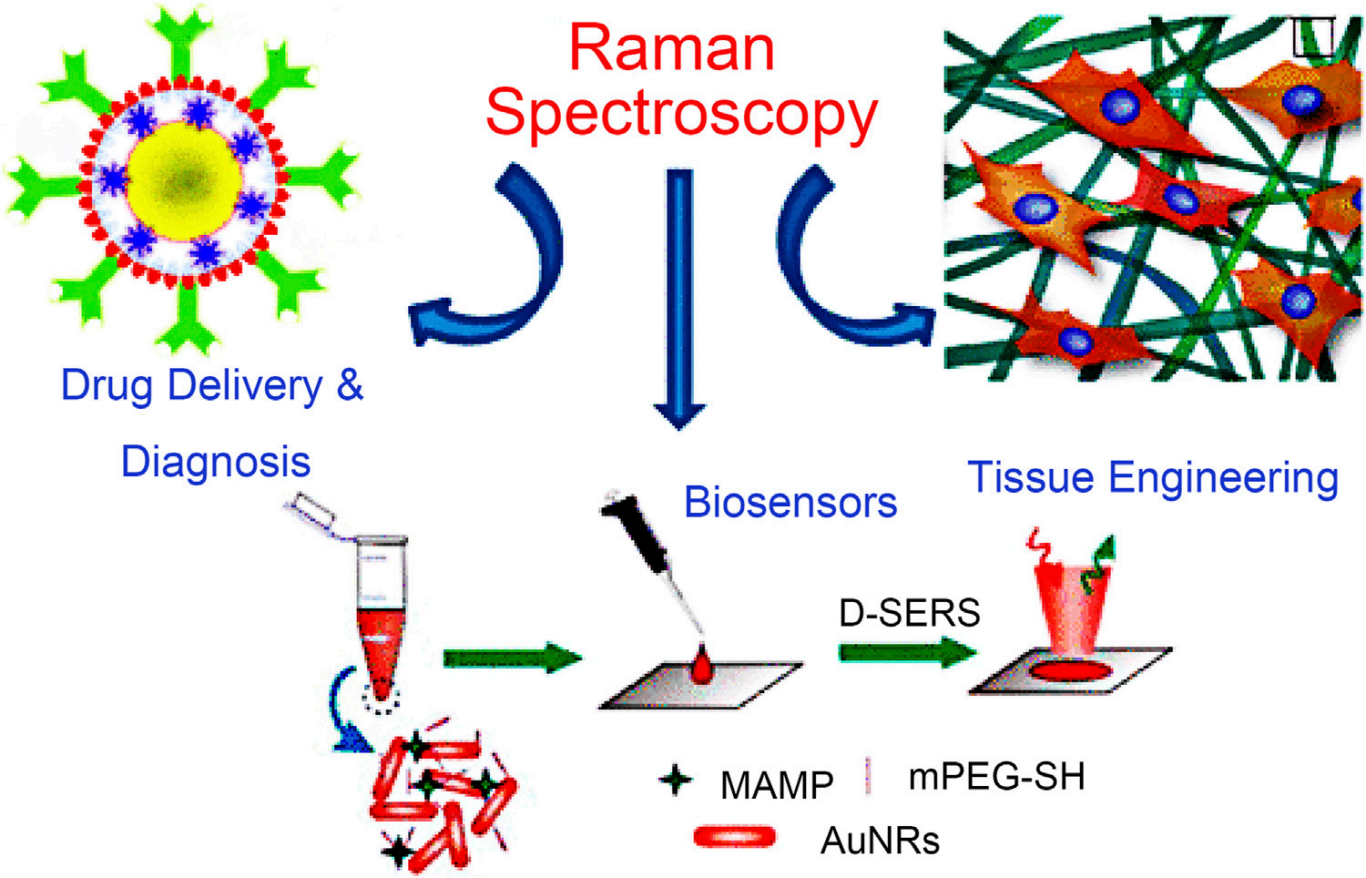
Application of Raman spectroscopy in drug tracking, biomarker detection, and cell engineering (Agrawal and Samal, 2018).

**FIGURE 2:**
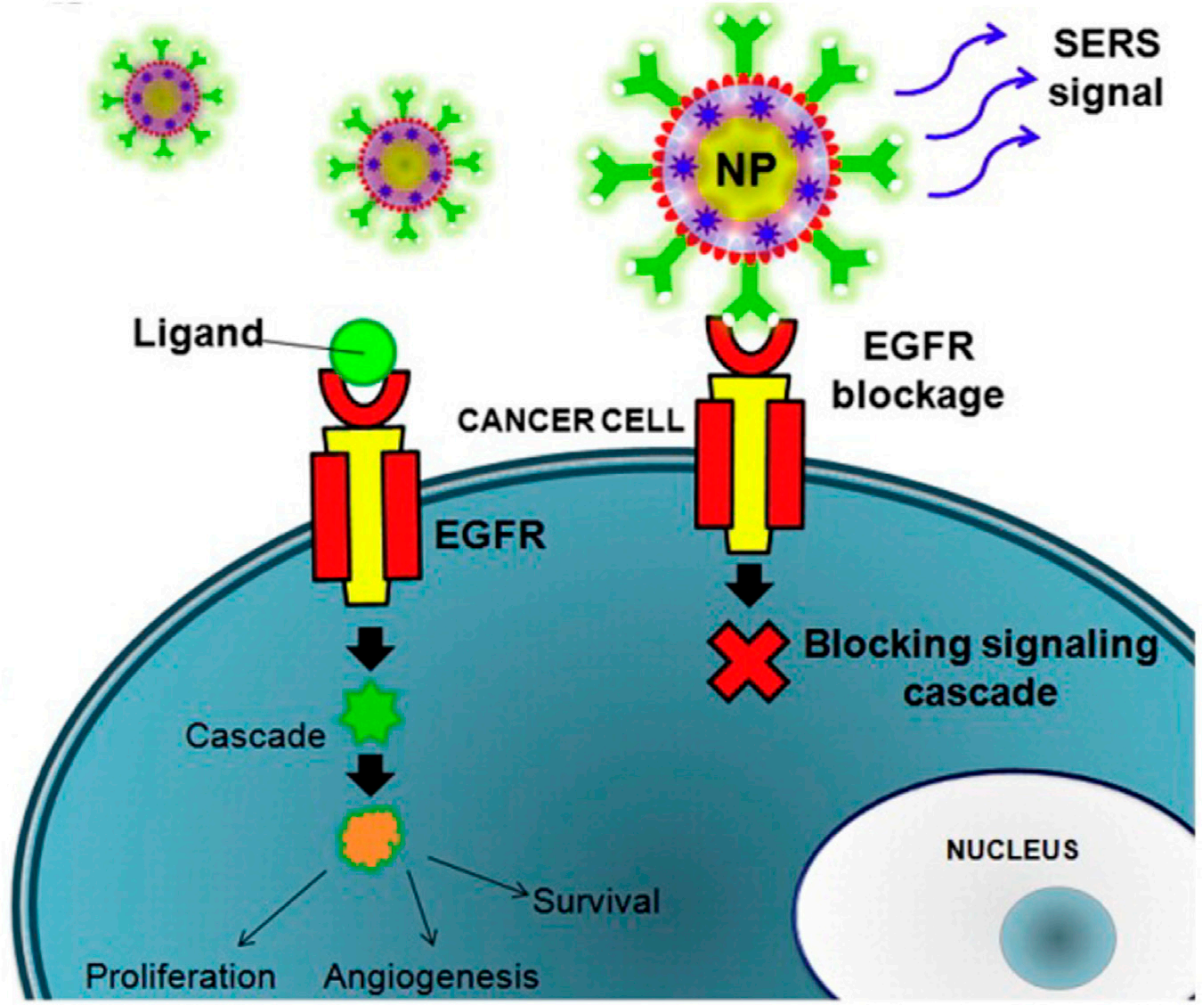
The combination of nanoantenna and cancer cells inhibits proliferation (Agrawal and Samal, 2018).

The Raman effect originates from the inelastic scattering of laser light, which can directly detect molecules and materials’ vibrational and rotational states (Jones et al., 2019). The basis of Raman scattering is the interaction between photons and molecules. The molecular transition energy can be obtained by measuring the wavelength of the Raman scattering photons. Therefore, the Raman spectrum is also called the chemical fingerprint spectrum. Raman spectroscopy has the advantages of being label-free and non-invasive, so it is widely used in life science research (Qiu et al., 2022). As shown in Figure 1, Raman spectroscopy technology has been used in drug tracking, biomarker detection, and cell engineering in various life science fields. The combination of nano antenna and cancer cell receptor inhibits cell proliferation. The SERS signal generated during this process may be identified by a Raman instrument and used to generate pictures for cancer cell phenotypic analysis. Numerous immune cells are involved in the tumor immunological response, increasing the detectable Raman spectroscopy level (Figure 2). Haematologic tumors have become a primary disease threatening human health, with 1.2 million new cases worldwide each year, and account for approximately 7% of all newly diagnosed cancers (Auberger et al., 2020). Haematologic malignancies are a group of highly heterogeneous diseases involving the blood, bone marrow, and organs. Common hematological malignancies include leukemia, myelodysplastic syndromes (MDS), malignant lymphoma (ML), and multiple myeloma (MM). Many rare gene mutations have been found to cause hematological tumours, such as acute leukemia and myelodysplastic syndromes. They differ in penetrance, age of onset, and clinical manifestations. Because of this heterogeneity, there is no unified standard diagnostic method for their diagnosis and treatment (Bochtler et al., 2018). In recent years, although there have been an increasing number of diagnostic procedures for hematological tumors, with the development of Raman spectroscopy and its wide application in many fields, its value in the diagnosis of tumor biology is also increasing (Huang et al., 2022).

In addition to its outstanding achievements in biochemistry, Raman spectroscopy is also highly known in liquid biopsy and epigenetic diagnosis. Abnormal methylation of gene promoters may be the inactivation mechanism of tumor suppressor genes. Studying cancer epigenetics and its key role in tumorigenesis through Raman spectroscopy can provide attractive epigenetic therapeutic targets and is a powerful tool for disease monitoring and prognosis estimation (Turcas et al., 2020). The new Raman spectroscopy development technology compensates for the drawbacks of classic Raman scattering, such as excessive noise and poor signal intensity. It has been further verified and developed in biological analysis applications, including single-cell Raman, surface-enhanced Raman, atomic force Raman, liquid biopsy, and other diagnostic and therapeutic methods, providing a new bright prospect for monitoring the occurrence and development of diseases, their prognosis, and treatment.

## Current status of the diagnosis of haematological tumours

Exploring tumor pathogenesis and therapy has recently been a popular research topic. Cell senescence is an essential mechanism in preventing the proliferation of potential tumor cells, but it can also promote tumor growth. Increasing evidence has shown that cell senescence is related to the pathogenesis and development of hematological malignancies, including leukemia, myelodysplastic syndromes (MDS), and multiple myeloma (MM). Cell senescence is associated with a decline in hematopoietic stem cell function and an increased risk of hematological malignancy (Hu et al., 2021). It is believed that hematological malignancies are only driven by genetic or epigenetic diseases in hematopoietic cells (Méndez-Ferrer et al., 2020). Most hematological malignancies are sporadic diseases; myeloid and lymphocytic lineage diseases with genetic susceptibility are related to heterogeneous clinical manifestations, and many symptoms are specific to certain cytogenetic and molecular aberrations (Kotmayer et al., 2020).

Histopathology is a standard method for evaluating hematological malignancies, but it has significant limitations in research and clinical settings. A patient’s bone marrow biopsy can fail to detect the heterogeneity of the disease, which may result in non-diagnostic samples; thus, this technique cannot be used repeatedly in clinical oncology (Robison et al., 2022). Malignant tumors use many strategies to escape the antitumor immunity of the adaptive immune system by creating an immunosuppressive microenvironment (Kasakovski et al., 2018). The new miRNA regulatory factors and pathways related to miRNA imbalance are related to hematological malignancies, and miRNA expression profile analysis is a crucial tool in predicting hematological malignancies and treatment responses (Han et al., 2020). A liquid biopsy is an essential tool for the diagnosis, patient stratification, and therapeutic monitoring of blood and solid cancer. However, due to the limited sensitivity of liquid biopsy samples, it is necessary to explore appropriate analysis methods (Bogeska, 2021).

CAR-T cell therapy is an effective new therapy for hematological malignancies. The dose level of CAR-T cells and the early and peak levels of cytokines are key indicators for diagnosing and treating hematological malignancies. The precise monitoring of this micro bioactive substance *in vivo* needs further research. Raman spectroscopy is highly sensitive and non-destructive. CAR-T cell therapy also places the utmost importance on the probe design of CAR-T cells. Combining Raman spectroscopy and nanoprobes has made this task easier. Raman spectroscopy as an adjunct to CAR-T cell therapy is a viable option (Brudno and Kochenderfer, 2019).

**FIGURE 3:**
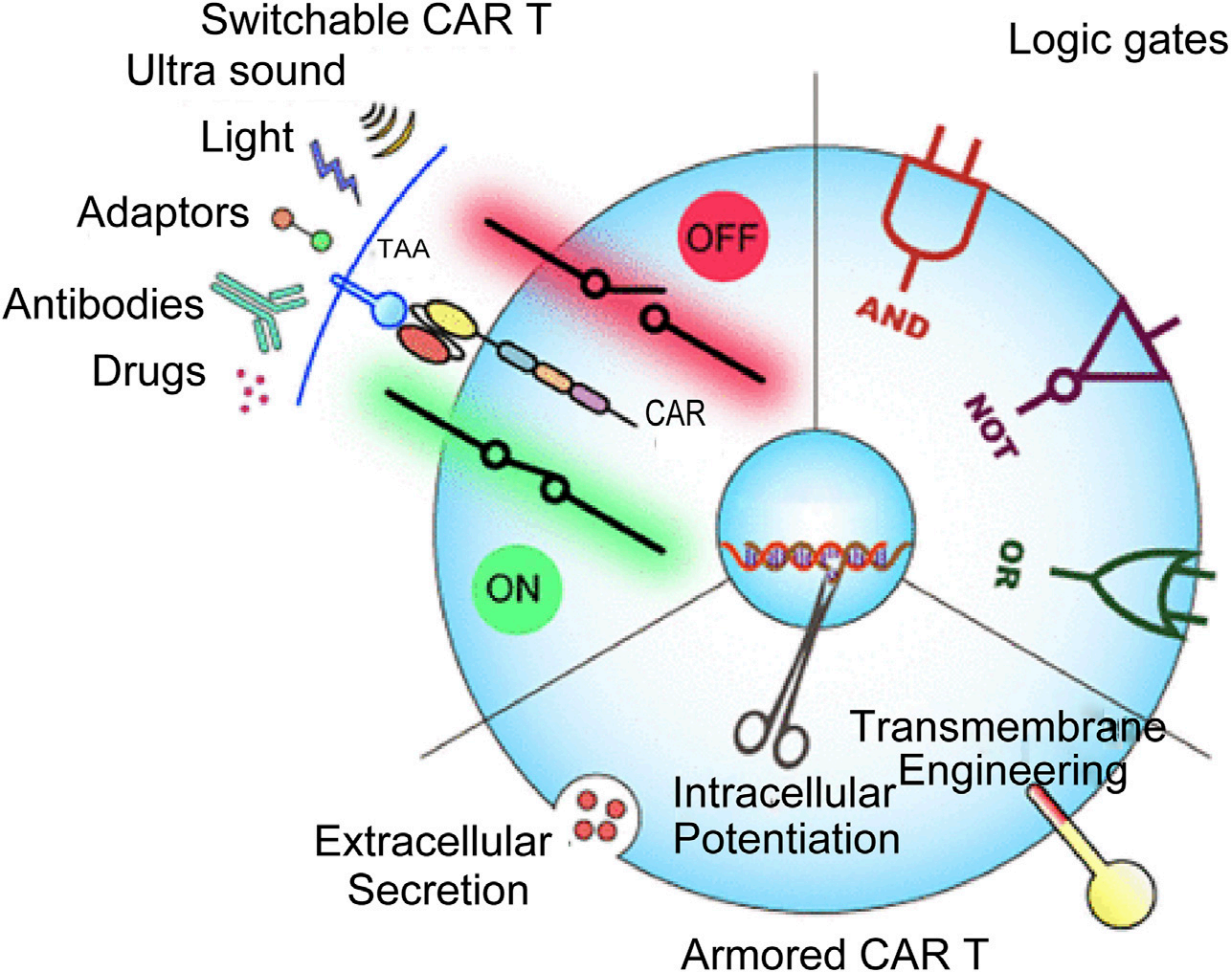
CAR-T-cell therapy in cancer treatment (Zhang et al., 2022).

Figure 3 illustrates the innovative new component of cancer treatment known as antigen receptor (CAR)-T cell therapy. CAR-T cells are sometimes called “redirecting T cells for universal cytokine-mediated death.” The extracellular antigen sensing domain, the extracellular hinge or spacer domain, the transmembrane domain, and the intracellular signal domain are the four major components of CAR-T. CAR-T cells use an external induction or self-regulating ON/OFF switch to improve safety and controllability. A combination of logic-gated T-cell activation recognizes the antigen to begin a cellular immune response. Although CAR-T cell treatment has significantly improved clinical outcomes in specific subgroups of B-cell leukemia or lymphoma, its therapeutic impact on solid tumors and blood cancers are limited for various. Some challenges faced by CAR-T cell treatment include severe toxicity, targeted cancer release effects, antigen escape, tumour transport, and restricted tumor penetration. In addition, CAR-T cells’ ability to function is severely hampered by the interaction between the host and tumor microenvironment. These therapies must also be developed and implemented by a sophisticated staff. Designing more potent CAR-T cells is necessary to meet these obstacles, enhancing antitumor efficacy and lowering toxicity (Sterner and Sterner, 2021). However, classic hematological tumor diagnostic and treatment impact evaluation approaches in clinical practice are time-consuming and expensive. They are invasive, and some disease types have no “gold standard” for diagnosis. Therefore, developing a quick method for identifying hematological tumors based on Raman spectroscopy without needing antibody labeling and the in-depth analysis of vast serological examination data of patients with hematological tumors is very important for the early detection of diseases. Additionally, this will lower detection costs, raise the development of speedy and accurate diagnosis and treatment of haematologic cancers, and enhance the diagnostic effectiveness and treatment level of diseases.

## Raman spectroscopy

Elastic and inelastic scattering are examples of scattering events that can happen when photons interact with materials (Dell'Olio, 2021a). Inelastic light scattering of molecules was first observed by the Indian scientist Raman in 1928 and later called Raman scattering. Today, Raman spectroscopy is a promising analytical tool that can provide chemical fingerprints for molecular identification. When photons interact with matter, the frequency of most scattered light will not change (Rayleigh scattering). However, inelastic light scattering under incident light may also occur; Raman scattering occurs when scattered light is emitted at a frequency greater or less than the incident light due to molecular vibration. The Raman spectrum comprises wavenumber bands, and each functional group of the sample molecule’s position in the range is determined by its particular vibration frequency (Serebrennikova et al., 2021). This method detects the molecular vibration and rotation related to the chemical bond in the sample to learn more about the molecular structure, composition, and intermolecular interactions. By observing the intensity distribution of inelastic scattering light as a function of frequency, it is possible to identify the distinct spectral fingerprints of different tissue samples. The spectra produced by nucleic acids, proteins, lipids, and carbohydrates with Raman-active functional groups may assess, describe, and differentiate different tissue types since each sample has a unique composition (Auner et al., 2018).

The developed technologies include surface-enhanced Raman spectroscopy (SERS), resonance Raman scattering (RRS), tip-enhanced Raman spectroscopy (TERS), and coherent anti-Stokes Raman scattering (CARS). According to the “hot spot” phenomenon at the intersection of dimers and clusters in metal nanoparticles, which is the basis of the SERS detection method, electromagnetic solid field augmentation is produced due to aggregation. The Raman scattering signal of the adsorbed molecules is strongly amplified to 14 orders of magnitude with a highly enhanced SERS substrate when molecules are adsorbed on the surface of rough metals (such as Ag, Au, or Cu) or metal nanoparticles (i.e., SERS substrate), allowing for the detection of a single molecule. SERS offers exceptionally high sensitivity and thorough fingerprint information and has developed into a potential DNA rapid identification and structural characterization method. SERS has the optimal resolution and can evaluate and classify complex spectrum data when used with chemometric techniques. Multiple target DNA detections are an important clinical application of SERS detection because of their simplicity, speed, and high sensitivity advantages. Even though SERS has great promise, its analytical performance still needs to be improved. The challenge in furthering this technology is creating a consistent process for uniformly producing SERS spanning various samples. In addition, the experimental design of clinical sample detection should be adjusted to obtain quantitative detection of analytes in complicated biological samples.

In humans, the level of 5-methylcytosine (5 mC) generated by cytosine methylation is regarded as a diagnostic biomarker for early cancers and a risk assessment tool for the clinical treatment of bacterial and viral infections. Using resonance excitation of the wavelength in the UV region, RRS modifies the excitation wavelength to the absorption resonance of the target 5 mC or unmethylated cytosine at the ring absorption resonance. Raman signals can be improved without tags, and the measurement pipeline is shorter because no amplification, library formation, or other operations are required.

TERS combines the chemical selectivity of the Raman spectrum with the spatial resolution (in nm) of the atomic force microscope (AFM) and scanning tunneling microscope (STM). The metal nanostructures on the AFM tip or STM tip itself (STM-TERS) alter the incident laser’s electromagnetic field and improve the Raman scattering of a few samples close to the tip. The tip of the TERS functions as a nanoantenna, which converts the electric field of the incident laser beam into local energy, thereby improving the spatial resolution of the TERS to a few nanometers under vacuum and low-temperature conditions and transforming the near field of the sample into a far-field accessible to the objective lens, thereby increasing the sensitivity to a single molecule. TERS has the benefit of observing the molecular alteration of many functional groups and DNA base pairs in the DNA skeleton as well as the development of new chemical bonds between DNA strands and various substances simultaneously. Despite many groundbreaking findings, TERS possesses excellent atomic resolution even in the strong coupling state. Many practical problems remain in cutting-edge improved nano spectroscopy’s easy and widespread deployment. TERS enhancement factors are not uniform for distinct plasma tips, even under the same excitation settings. Since their vertex size, shape, and surface roughness are slightly different, controlling them at the nanoscale is difficult. Similarly, maintaining the vector field and mode curve at the apex remains difficult.

A type of unregistered non-linear chemical imaging technique called a coherent anti-Stokes Raman scattering (CARS) microscope might identify particular inherent molecular vibrations in samples. The vibration resonance of the target molecules is matched by the frequency difference between two laser beams. Without fluorescent labeling, the excitation laser in biological imaging can be tuned to resonate with the vibration of lipids, proteins, and even DNA. Living cell imaging has extensively used coherent Raman imaging, which has a high collection rate and excellent sensitivity for detecting the presence of lipids, proteins, nucleic acids, and water in cells. The sensitivity of CARS is still at the millimole level of intrinsic biomolecules despite these benefits and qualities. CARS microscopy has difficulty achieving single molecule level measurement without other enhancing techniques.

The biological application prospect of Raman spectroscopy is broad. Real-time detection, *in vivo* detection, and biomarker detection may increase the accuracy of diagnosing precancerous lesions, aiding clinical screening, symptomatic treatment, and prognosis development (Upchurch et al., 2018). SERS has high sensitivity and excellent multiple detection ability. It provides a more precise method for diagnosing myelodysplastic syndromes by detecting specific biomarkers to differentiate the development stage of red cells from bone marrow hematopoietic stem cells. SERS can also be used for gene detection and point mutation site identification, providing a basis for the molecular biological diagnosis of myelodysplastic syndromes. SERS validates molecular detection targeting functions based on nanotags. The high load and rich surface modification methods of nanomaterials provide a variety of possibilities for improving biological compatibility, pharmacokinetics, and drug targeting of drugs. Nanoparticles have unique physical and chemical properties, and biological characteristics, The combination of SERS and SERS has advantages in diagnosing and treating MM tumors (Huang et al., 2020). SERS combines traditional Raman spectroscopy’s structural specificity and experimental flexibility with the ultra-high sensitivity of the signal amplification mediated by plasma nanostructures. It provides the possibility of simultaneously detecting multiple biomarkers in body fluids and obtaining the internal fingerprint information of biomolecules, which can selectively identify the antibodies of biomarkers and improve the detection capability. It is instructive for MM who have not yet received precision medical treatment (Zhang et al., 2019).

By combining the chemical selectivity of the Raman spectrum with the spatial resolution (nm) of AFM, STM, and other technologies, TERS can analyze the molecular modification of several functional groups and DNA base pairs in the DNA skeleton and detect the formation of new chemical bonds between DNA chains and various chemical substances. Clinical optical biopsies can benefit from TERS’s analysis of dynamic response changes in living cell molecules, allowing for more precise identification of different lymphoma cell types. Raman spectroscopy and RESPECT probe fingerprints compare the relative peak differences between non-Hodgkin’s lymphoma subtypes, providing diagnostic and therapeutic targets. Single-cell Raman spectroscopy has; high resolution, non-destructive, molecular specificity, and independent culture advantages. It can analyze the potential molecular differences between different leukocyte subtypes for leukemia diagnosis and detect the metabolites of leukemia cells without causing damage; when combined with flow cytometry, single-cell metabolic phenotypes can be monitored. The differential diagnosis technology of CLL and DLBCL was developed by non-invasive detection of plasma. Analyzing the 1655cm^−1^ Raman peak to study the changes in prognosis-related hemoglobin and serum albumin is beneficial to ML’s diagnosis and prognosis monitoring (Table 1).

**TABLE 1:** Summary of different Raman methods as diagnostic tools for different tumors.

Raman spectroscopy	Characteristics	Advantages	Disadvantages	Applications
Surface-enhanced Raman spectroscopy	An enhanced signal on the metal substrate	High sensitivity and multiple detections	Less choice of substrate and difficult control of stability	Diagnosis of the myelodysplastic syndromes; Diagnosis of multiple myeloma; Liquid biopsy
Tip-enhanced Raman spectroscopy	Combined with scanning probe microscopy	High sensitivity, high resolution, unrestricted substrate	Easy to generate false signals under the influence of pollutants, and the sample is decomposed	Lymphoma diagnosis; Clinical optical biopsy
Single-cell Raman spectroscopy	Single-cell imaging analysis	High resolution, no damage, and small sample size; can be used to determine single-living cells	The technology is not perfect and has not been combined with downstream sequencing and culture technology	Diagnosis, classification, and prognosis monitoring of lymphoma; Diagnosis of leukemia
Optical tweezers Raman spectroscopy	Study on a single cell in suspension with laser tweezers	Fast, non-destructive, highly specific, and sensitive	High requirements for laser	Diagnosis and evaluation of prognosis of leukemia Drug resistance

**FIGURE 4:**
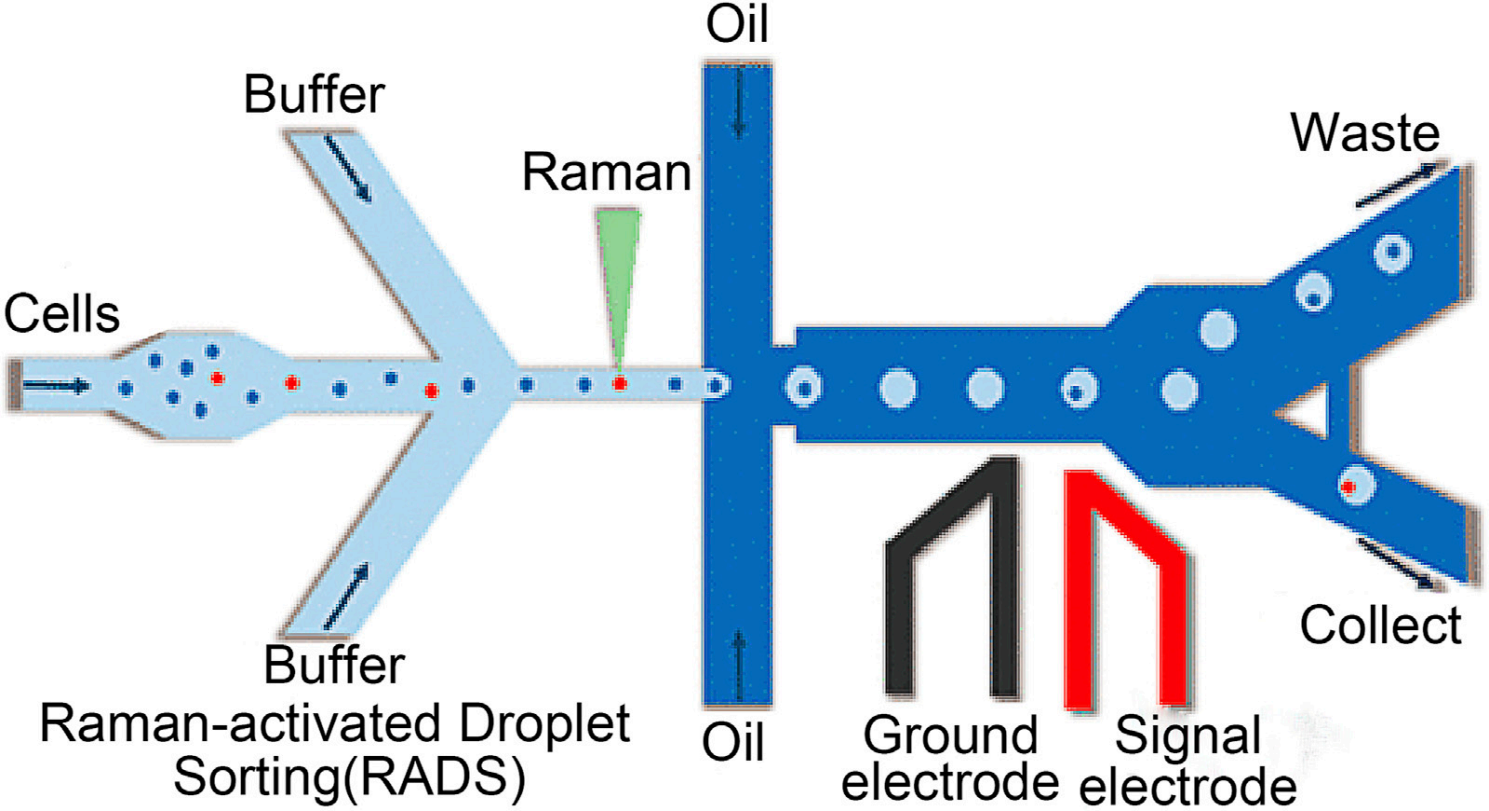
The process of Raman living cell sorting (Wang et al., 2017).

With its high sensitivity and compatibility with *in vitro* samples, Raman spectroscopy is becoming increasingly popular for obtaining chemical images of cell samples in parasitology, pharmacology, and oncology (Durrant et al., 2019). Raman spectroscopy has also been widely used to diagnose diseases *in situ* (Pence and Mahadevan-Jansen, 2016). Modern handheld and portable Raman spectrometers, which are frequently used in drug quality screening, have good repeatability, can detect low concentrations of substances in mixtures of several component substances, or can, with the help of suitable mathematical processing techniques, distinguish between compounds with similar structures and slight spectral differences (Galeev et al., 2019). Zhao et al. created a multiplex Raman probe panel with sharp and mutually resolvable Raman peaks. The panel demonstrates the viability of this method in living-cell analysis and phenotypic characterization under diverse drug disruptions when combined with whole-cell spontaneous Raman spectroscopy. Figure 4 shows the process of Raman living cell sorting. In order to obtain and analyze Raman spectra, buffer solution and cell suspension are added during the Raman sorting process of living cells; The cells were collected and individually encased into droplets; Droplets that are not targeted automatically flow into the garbage channel. Triggered electrode sorting divides the target droplets into “collection” channels.

Because Raman spectroscopy provides unique molecule-specific information, it has great application potential in researching various diseases (Fales et al., 2021). An essential advantage of Raman spectroscopy is that it usually requires little or no sample preparation and can directly collect data from various sample types. In addition, Raman spectroscopy can provide a reasonable penetration depth to the sample so that thicker or complete tissues can be evaluated. Contrary to the infrared spectrum, the water signal in the Raman spectrum is usually weak, which avoids the interference of the water zone when analyzing highly hydrated tissues (Querido et al., 2021).

**FIGURE 5:**
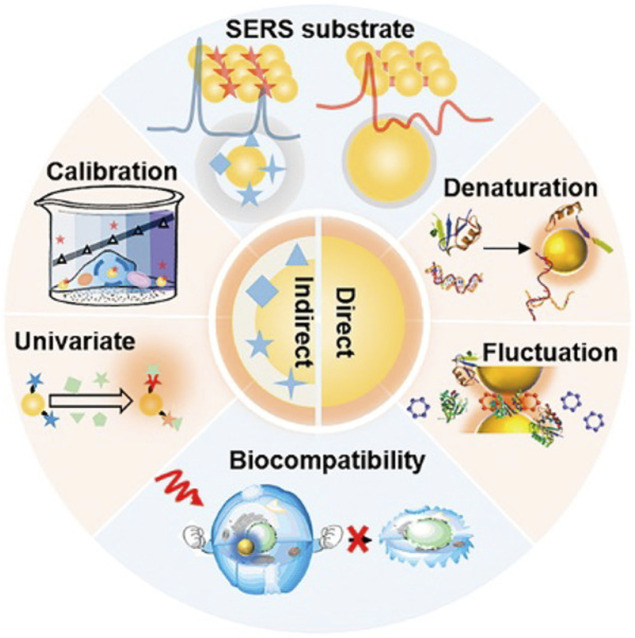
Application of surface-enhanced Raman spectroscopy in biology (Zong et al., 2018).

For the accurate identification and treatment of blood malignancies, it is still difficult to monitor the smallest residual of medications and cancer cell secretions. SERS, however, has emerged as a promising therapeutic technique to do this. The Raman scattering signal of the adsorbed molecules is strongly amplified to 14 orders of magnitude with a highly enhanced SERS substrate when molecules are adsorbed on the surface of rough metals (such as Ag, Au, or Cu) or metal nanoparticles (i.e., SERS substrate), allowing for the detection of a single molecule. SERS has been widely employed in various applications, including chemical analysis, biomolecular detection, biological agent diagnosis, DNA sequence, environmental monitoring, and others because of its unmarked fingerprint recognition, high sensitivity, and quick detection capabilities. In addition, the standardization, quality assurance, and technical preparation of self-assembly methods and nano-manufacturing procedures have been rigorously assessed. Bonifacio et al. demonstrated that SERS can be a powerful tool for developing care point testing of therapeutic drug monitoring (TDM) for drugs with narrow therapeutic windows. The use of SERS in the biological analysis is shown in Figure 5. SERS for biological investigation has been a rapidly expanding study area in recent years. SERS is an efficient analytical technique anticipated to have exceptional potential in biological analysis and diagnosis as it is *in vivo* detection projects develop. This is due to its high sensitivity and good multiple detection capabilities. Based on direct and indirect methods, SERS enables the quick identification of molecular species. It offers a wide variety of therapeutically relevant applications, including biosensors, drug administration, and live cell imaging, since it uses the adaptable surface characteristics of nanostructures (Tahir et al., 2021). The SERS can be applied to the diagnosis of various hematological tumors, including cancer tissue identification and tumor boundary division, the detection of changes in basal cells after treatment, cancer-specific exosomes characterization, and cancer cell differentiation from non-cancer cells (Grieve et al., 2021).

Single-cell Raman spectroscopy (SCRS), which has grown more complicated *via* integration with other cutting-edge analytical technologies and contemporary data processing, can now explore complex biological and environmental samples in a way that has never been possible with considerably better specificity, sensitivity, and resolution. SCRS-derived technology is superior to conventional batch measurement in environmental and biological research because of its high resolution, label-free and non-invasive nature, molecular specificity, culture independence, and application to *in situ*, *in vitro*, or *in vivo* studies (Wang et al., 2020). Some studies have used non-label and non-invasive SCRS techniques to characterize the molecular cell spectrum. A group of Raman peaks can be found to identify myeloblasts, abnormal promyelocytes, and normal granulocytes, proving that Raman spectroscopy detects the metabolites of leukemia cells non-invasively. The biomarkers identified in this study can be extended to other blood cells and may significantly impact leukemia treatment (Cheng et al., 2022).

The interference of the laser-induced fluorescence signal, which often spans the scattering band, is the fundamental drawback of Raman spectroscopy. Another significant drawback is the sample’s inherent spontaneous fluorescence. Because specific molecules and contaminants in the extracellular matrix may interact with input light to produce fluorescent photons with greater efficiency than scattered photons, interfering with the Raman spectrum, this is a particular difficulty in the interior environment of biological tissues (Cortijo-Campos et al., 2021). Raman signals from biological tissues are usually weak, and the signal-to-noise ratio of the spectrum will also affect the accuracy of the measurement (Wróbel et al., 2021). Raman spectroscopy shows promising results in medical diagnosis by providing unmarked and precise molecular information of pathological tissues *in vitro* and *in vivo*. However, the high specificity of Raman spectroscopy comes at a cost. The acquisition rate is low, depth information cannot be directly accessed, and the sampling area is limited (Schie et al., 2021).

### Application of Raman spectroscopy in leukemia

Leukemia is a group of heterogeneous hematological diseases characterized by invalid hematopoietic cells and morphological abnormalities (Shahrabi et al., 2018). Leukemia has a complex diagnostic process, leading to a high mortality rate when a diagnosis is not obtained on time. The main signs and symptoms of patients with symptoms not found by routine blood examination and patients with serious diseases due to sepsis in the case of severe bone marrow failure may be different. The early manifestations of the disease are non-specific. It is essential to evaluate the medical history, social history, and overall health status of patients with leukemia in the early stages because these factors play a vital role in treatment decision-making (Newell and Cook, 2021). Cytogenetic analysis and clinical application of molecularly targeted drugs have significantly improved the prognosis of many patients with hematological malignancies, especially those with chronic myeloid leukemia and acute promyelocytic leukemia. However, treating hematological malignancies still faces problems such as disease recurrence and drug resistance, so it is urgent to explore other potential molecular mechanisms (Xiao et al., 2020). Raman spectroscopy is utilized to determine possible molecular variations between leukocyte subtypes from genetic studies to diagnose leukemia. We can evaluate the molecules with biochemical changes in leukemia patients by detecting the spectral differences between whole blood and plasma samples from healthy and leukemia patients. We can also identify the distinctive spectroscopic markers of leukemia cells using single-cell imaging technology. Raman spectroscopy is used to establish a prognosis model, predict the prognosis of leukemia, study the molecular biological differences between patients with a good or bad prognosis and healthy individuals, predict the drug resistance of leukemia cell therapeutic targets, and further observe the prognosis of leukemia by looking for genetic abnormalities and mutations.

**FIGURE 6:**
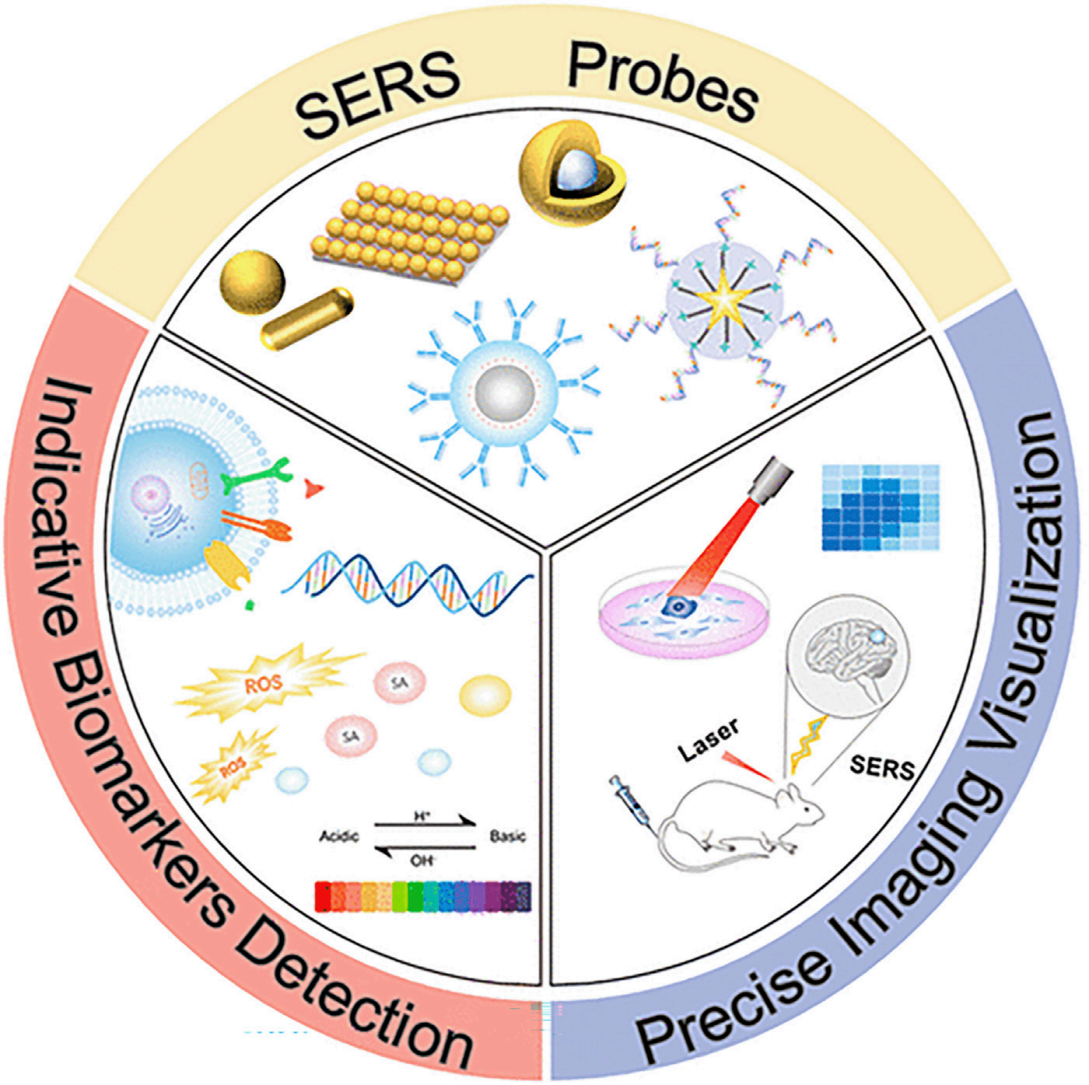
Single-cell Raman imaging technology for detecting the microenvironment of disease-related proteins, nucleic acids, and small molecules (Lin et al., 2021).

Li et al. used low-frequency wavelet coefficients to reconstruct the spectrum based on the coverage of segmented spectral data at 720–800 cm^−1^. They carried out the multicomponent analysis, which can better identify the potential molecular differences between leukocyte subtypes without labeling (Li et al., 2020). Silva et al. suggested using dispersive Raman spectroscopy to determine the spectral differences between whole blood and plasma samples from healthy and leukemia subjects, correlate these differences with their biochemical changes, perform discriminant analysis on the samples (n = 38 whole blood and n = 40 plasma samples), and use a Raman probe to obtain the Raman spectrum. Exploratory research using spectral principal component analysis of blood and plasma samples revealed that the healthy group’s peak value for proteins, amino acids, free phospholipids, and carotenoids was greater than that of the leukemia group (da Silva et al., 2018). Leszczenko et al. used single-cell Raman imaging and multivariate statistical analysis to separate the cells from the bone marrow of individuals with three subtypes of B-cell precursor acute lymphoblastic leukemia. The single-cell B cell spectrum from healthy donors was compared to the range obtained from clinical samples to create a control group. Studies have shown that, particularly in band strength, the Raman spectra of normal B cells and those of their malignant counterparts are considerably different. Using chemical metrology can automatically identify leukemia subtypes, verifying the clinical applicability of Raman imaging in identifying leukemia cell characteristics and sub markers (Leszczenko et al., 2021). As shown in Figure 6, in clinical diagnosis, SERS-assisted single-cell Raman imaging technology can detect the microenvironment of disease-related proteins, nucleic acids, and small molecules, which can be used to assist in medical diagnosis.

Tongs with a laser Raman spectroscopy (LTRS) combine laser tweezers and Raman spectroscopy. It is a physical instrument based on the laser mechanical effect that may be used to swiftly and non-destructively investigate individual live cells in suspension. To promptly and non-destructively identify and assess the treatment resistance of acute lymphoblastic leukemia (ALL) cells, Chen et al. developed a novel approach based on LTRS. The research contained two distinct doxorubicin-resistant parental ALL cells, BALL-1 and Nalm6. Raman spectrum variations produced by adriamycin-resistant cells may be seen using LTRS. Chen et al. demonstrated that the specificity and sensitivity of LTRS are more than 90% by using principal component analysis (PCA) and classification and regression (CRT) methods to assure the correctness of the findings. In addition, to further clarify the chemical resistance state of all cells, Chen et al. used the CRT model based on strip data and receiver operating characteristic (ROC) curve analysis to identify essential strips and strip intensity ratios with solid directional significance. Experiments have proven that the combination of LTRS analysis and multivariate statistical analysis has great potential and can become a new analytical strategy for the rapid evaluation of the drug resistance status of all cells at the single-cell level (Chen Y et al., 2022).

The excessive synthesis of aberrant B-cell lymphocytes from bone marrow results in chronic lymphocytic leukemia (CLL). Slowly worsening CLL is an incurable condition. Static CLL-related cells may be seen in the bone marrow, lymph nodes, spleen, and blood. CLL cells must be highly adaptive to oxygenation and molecular cues from the matrix due to the variety of microenvironments in which they are found. For instance, lymph nodes’ non-CLL cells’ cell signal transduction affects CLL cell growth differently than elsewhere. In addition, the microenvironment in the bone marrow of CLL patients is characterized by the increased expression of mRNA and protein levels of the BCL-2 protein family, which is the crucial anti-apoptotic factor that helps maintain CLL proliferation and the basis of its drug resistance tendency so that it can be used as a therapeutic target (Nemkov et al., 2019). Amini et al. used tetracationic ditriarylborane 1,3-butylene as a fluorescence and Raman probe, which can simultaneously and selectively detect various DNA, RNA, and proteins (Amini et al., 2020). The SERS-based technique was developed by Wang et al. to quickly and accurately predict the outcome of acute myeloid leukemia (AML) following treatment. The molecular biological differences between AML patients with excellent and poor prognoses and those without AML were examined using SERS measurement of bone marrow supernatant samples. Multivariate analysis of SERS readings was conducted to construct a predictive model of AML. The findings revealed that AML patients with excellent and dire prognoses had significantly different amounts of amino acids, carbohydrates, and lipids. The AML prognostic model’s prediction accuracy was 84.78%. The approach by Wang et al. has the potential to develop into a diagnostic tool for the prompt and precise prediction of the prognosis of AML (Chen S et al., 2021). AML is a biologically complex, molecular, and clinically heterogeneous disease. Its incidence increases with age. Cytogenetic abnormalities and mutation detection are essential prognostic tools for customized treatment after induction therapy (Medinger et al., 2019).

For acute lymphoblastic leukemia, gene analysis has long been an essential part of the diagnosis and treatment program for children and adults (Hellström Lindberg et al., 2021). Some Raman peaks associated with DNA showed systematic suppression at both dosage levels, according to Lasalvia et al. Even at the lowest dosage investigated, the intensity of the Raman peak at 784 cm^−1^, associated with the DNA phosphate group stretching mode, is very sensitive to proton beam exposure. As a result, it may be used to diagnose genetic abnormalities and mutations in AML and as a spectral marker of cytogenetic damage (Lasalvia et al., 2019).

### Application of Raman spectroscopy in MDS

Acute myeloid leukemia is more likely to develop in people with myelodysplastic syndromes (MDS), a set of various myeloid disorders marked by peripheral blood cell loss. Men over the age of 50 and those who have had cytotoxic treatment in the past are more likely to develop myelodysplastic syndromes. At the bone marrow sample and gross inspection time, morphological evidence of dysplasia was used to diagnose MDS. The comprehensive diagnostic analysis of information usually obtained from karyotype, flow cytometry, molecular genetics, and other studies has certain limitations in the clinical application (Garcia-Manero et al., 2020). To diagnose the condition, Raman spectroscopy was utilized to examine the unique characteristics of bone marrow mesenchymal stem cells from myelodysplastic syndromes patients. Specific biomarkers were found to differentiate between the cell development stage of bone marrow hematopoietic stem cells to red cells to diagnose the myelodysplastic syndromes, using anemia performance as the entry point. In terms of treatment prognosis, Raman spectroscopy was used to analyze the chemical differences between CD4 and CD8 T cells and to observe the occurrence of autoimmune diseases through the changes of T cells because AD frequently occurs in young patients with a high international prognosis risk score. 80% of patients had single or multiple gene mutations, and SERS can accurately identify point mutation sites to diagnose myelodysplastic syndromes rapidly. Raman spectroscopy was utilized by Kukolj et al. to examine the individual variations among bone marrow mesenchymal stem cells. Raman spectroscopy was used to analyze bone marrow mesenchymal stem cells at the single-cell level. The results revealed that each donor’s bone marrow mesenchymal stem cells had slightly different Raman spectra despite having a comparable metabolic background. The research found that this technique may diversify the bone marrow mesenchymal stem cell population after doing a thorough principal component analysis of Raman spectroscopy. Raman spectroscopy, an unmarked measuring technique, offers significant promise for understanding stem cell heterogeneity and grouping cell populations with comparable biochemical backgrounds, which is crucial for creating individualized MDS treatment approaches (Kukolj et al., 2022). Myelodysplastic syndromes (MDS) originates from hematopoietic stem cells but has very heterogeneous biological and genetic characteristics. The main clinical feature is haematopenia, and the risk of progression to acute myeloid leukemia is high.

Although MDS includes heterogeneous subclasses, they share a common origin in hematopoietic stem cells and progenitor cell compartments. The degree of haemocytopenia partly determines the subtypes of MDS, but some MDS and mixed MDS/myeloproliferative tumor (MPN) subgroups may show increased leukocyte, monocyte, and platelet counts. In addition, patients with mild or marginal anemia can be diagnosed with early MDS if there are apparent morphological or cytological changes (Hellström-Lindberg et al., 2020). Anemia is the most common clinical manifestation of myelodysplastic syndromes (MDS), and most patients rely on red blood cell infusion for treatment. Defective erythropoiesis includes the impairment of terminal erythrocytic maturation. If red blood cell stimulation drugs fail, the treatment options for low-risk MDS anemia are limited (Komrokji, 2020). Alattar et al. proved that SERS could determine the cell development stage of bone marrow hematopoietic stem cells from red cells by identifying specific biomarkers of SERS. The method adopted allows the use of gold nanoparticles as Raman-enhanced substrates for dynamic structure observation of cells through multidimensional parameters to obtain bone marrow hematopoietic stem cells from proliferating (phase I), differentiating (phase II) and mature red blood cells (phase III) (Alattar et al., 2018). Autoimmune disorders (ADs) are seen in 10%–20% of patients with myelodysplastic syndromes (MDS).

The available data show that ADs more often occur in young patients with a higher risk of international prognosis score. The MDS subtypes associated with AD are mainly MDS-SLD and MDS-EB with excessive protocells (Grignano et al., 2018). T lymphocytes (T cells) are an essential part of the adaptive immune system and the key to understanding host responses to invading pathogens or pathogen-related molecular patterns. In the study by Ramoji et al., non-invasive Raman spectroscopy, as a label-free method, was used to track the changes in LPS-induced T cells in acute and acute inflammatory stages (1, 4, 10, and 30 days), especially CD4 and CD8 T cells in endotoxin C57BL/6 mice. Raman spectroscopy revealed the most significant chemical difference between CD4^+^ and CD8^+^ T cells from the control group and LPS-treated mice during acute inflammation. The difference could be seen 10 days after LPS injury. At the post-acute stage, CD4^+^ and CD8^+^ T cells from treated and untreated mice could not differentiate again, indicating that the T cells had largely recovered their original state. The biological information obtained from Raman spectroscopy is consistent with the immune reading, meaning that Raman spectroscopy is a very suitable label-free method that can be used to track the activation of splenic T cells in systemic inflammation from the acute to chronic stages to track the changes in the autoimmune system in some MDS patients (Ramoji et al., 2019). The early stage of MDS is characterized by hypoplasia of mature cells in peripheral blood or erythropoiesis in bone marrow, granuloma formation, or megakaryopoiesis. The late stage tends to present with the accumulation of primitive cells. Chromosome abnormalities are common in 50% of patients, and single or multiple gene mutations are common in 80% of patients. Chromosome abnormalities and gene mutations are the leading causes of abnormal differentiation and the accumulation of primitive cells in bone marrow (Giagounidis, 2020). Using SERS, Zeng et al. correctly located the point mutation sites and adequately analyzed the anti-single-strand DNA’s base composition and DNA frameshift mutation. This innovative approach can potentially be useful in gene diagnostics since it not only has a straightforward experimental procedure but also can precisely capture variations in the strength of the base ring peak induced by various nearby bases (Zeng et al., 2021). The application of SERS has dramatically promoted the efficiency of gene mutation diagnosis and provided a shortcut for the molecular biology diagnosis of MDS.

### Application of Raman spectroscopy in lymphoma

A lymphoma is a group of lymphocytic malignancies with more than 90 subtypes. Lymphoma usually presents painless lymph node enlargement accompanied by fever, unexplained weight loss, night sweats, and other systemic symptoms, which occur in the later stage of the disease (Lewis et al., 2020). Depending on the histological subtype, lymphoma cells express chemokine receptors and adhesion molecules differently. The pattern of surface molecules determines the location of a tumor’s involvement. Typically, stromal cells and macrophages, which are non-tumor cells, send signals to lymphoma cells by direct cell contact and paracrine factors (Nishikori, 2019). Over time, cytogenetic and molecular genetic techniques have been developed. Flow cytometry and immunohistochemical immunophenotypes combined with immunohistochemistry can accurately identify, diagnose and subclassify lymphoma and evaluate its abnormalities (Ferry, 2020). The use of flow cytometry as a novel biological analytical tool has been shown in the domains of metabolic engineering, cancer biology, stem cell biology, immunology, microbiology, and virology. It can quickly count and characterize numerous heterogeneous cells, including blood cells, stem cells, cancer cells, and microorganisms, as well as dissociated solid tissues, including lymph nodes, spleens, and solid tumors, in a suspension and provide a biological analysis of the cells by measuring cell size, cell particle size, and the expression of molecules on the cell surface and inside the cell. Because it primarily depends on fluorescent labeling for cell phenotypic characterization, which is an indirect assessment of intracellular chemicals and surface antigens, traditional flow cytometry has several significant drawbacks. It often requires lengthy preparatory processes and is incompatible with cell treatment.

To overcome these difficulties, different types of flow cytometry-based on Raman spectroscopy for the direct measurement of intracellular molecules have emerged, collectively called “Raman flow cytometry” for short. Raman flow cytometry can yield the chemical fingerprints of cells in a non-destructive manner and monitor the single-cell metabolic phenotype (Gala de Pablo et al., 2021). Raman spectroscopy and flow cytometry diagnose lymphoma by detecting the single cell metabolic profile and aberrant B-cell differentiation. When performing a clinical optical biopsy, TERS evaluates the dynamic behavior of biomolecules in living cells to distinguish between several types of lymphoma cells. When comparing non-Hodgkin’s lymphoma subtypes, Raman spectroscopy and RESpect probe fingerprinting show comparable peak differences. The Raman spectrum peak revealed that 1655cm^−1^ was connected to the changes in prognosis-related hemoglobin and serum albumin, which could be used as a prognostic indicator for treatment.

In response to T-cell-dependent antigens, mature B cells are stimulated to form germinal centers (GCs), which are also normal cells in most B-cell non-Hodgkin’s lymphomas. Although they share the same GC B-cell precursor, these lymphomas originate from cells at different stages of GC reaction and develop through other pathogenic mechanisms (Holmes et al., 2020). Monitoring the aberrant differentiation of B cells in great detail is crucial to understanding the lymphoma process. In biology and medicine, atomic force microscope (AFM) is a powerful tool for manipulating and analyzing live cells *in vitro*. Shibata studied the effects of indentation speed on the membrane perforation of living HeLa cells based on an atomic force microscope probe that catalyzes the highly localized photochemical oxidation of titanium dioxide. He also studied the photocatalytic nano processing and intracellular Raman imaging of living cells using functionalized AFM probes (TiO). Quantitative measurements were made of the likelihood, penetration force, and viability of cell membrane perforation using the force-distance curve acquired during the indentation process. They also looked at the potential for visualizing cells using TERS. This imaging is functionalized by using silver nanoparticles in a home-built Raman system combined with an inverted microscope through AFM probes, which successfully demonstrated that TERS imaging is capable of visualizing noticeably different features in the Raman spectrum between the nucleus and cytoplasm of a single living cell and of analyzing the dynamic behavior of biomolecules in living cells (Shibata et al., 2020).

In addition, Rau et al. studied lymphoid tissues collected from 20 patients who underwent surgery for suspected malignant tumors and collected approximately 400 μm^2^ tissue area images containing thousands of Raman spectra. Partial least squares discriminant analysis was used to create a lymph node classification model to distinguish benign and malignant tissues and to distinguish cancer type, grade, and BCL2 protein expression. This study provides a new idea for developing a clinical optical biopsy tool for diagnosing lymph node cancer using Raman spectroscopy (Rau et al., 2019). Garcia et al. analyzed pediatric non-Hodgkin’s lymphoma tissues and non-malignant specimens. An aluminum mirror-coated glass slide was used to evaluate the cryopreserved tissue using Raman spectroscopy and a RESpect probe. The RS sequential acquisition for the RESpect probe (EmVision, LLC, Loxahatchee, Florida) employs a unique two-component convergent lens that overlaps the laser excitation and collecting cones. The 785 nm laser (Watch Photonics, Durham, NC) is part of a more advanced RS portable system to which the RESpect probe is attached. This system is managed by a customized LabView (NI) program and evaluated using Enlighten software. The standard RS data from the laboratory RS instrument was used to prepare the RESpect probe utilizing the same tissue.

The similarity between non-Hodgkin lymphoma subtypes was revealed, and the typical Raman spectra and RESpect probe fingerprints showed comparable prominent peaks. Raman spectrum fingerprints and pediatric non-Hodgkin’s lymphoma subtypes and follicular hyperplasia peaks provide a new way to seek diagnostic methods and identify potential therapeutic targets (Agsalda-Garcia et al., 2020). To create a simple plasma detection system for non-invasive DLBCL and CLL detection, Bai et al. used Raman spectroscopy to examine the plasma features of individuals with diffuse large B-cell lymphoma (DLBCL) and chronic lymphocytic leukemia (CLL). 33 DLBCL patients, 39 CLL patients, and 30 healthy volunteers’ plasma were examined, and two groups were created using orthogonal partial least squares discriminant analysis (OPLS-DA). The DLBCL/CLL and control groups have essentially little overlap. The sensitivity and specificity for the CLL model were 92.86% and 100%, respectively, whereas, for the DLBCL model, they were 80% and 92.31%. DLBCL and CLL patients had distinct Raman bands in comparison to healthy individuals. The 1,445 cm^−1^ and 1,655 cm^−1^ Raman peaks could be used to distinguish DLBCL from CLL. Further analysis of the 1,655 cm^−1^ peak revealed changes in hemoglobin and serum albumin, potentially essential variables related to the prognosis of CLL. Raman spectroscopic plasma analysis has been shown to offer enormous promise as a novel clinical technique for the non-invasive identification of DLBCL and CLL (Bai et al., 2020).

### Application of Raman spectroscopy in other hematological tumours

**FIGURE 7:**
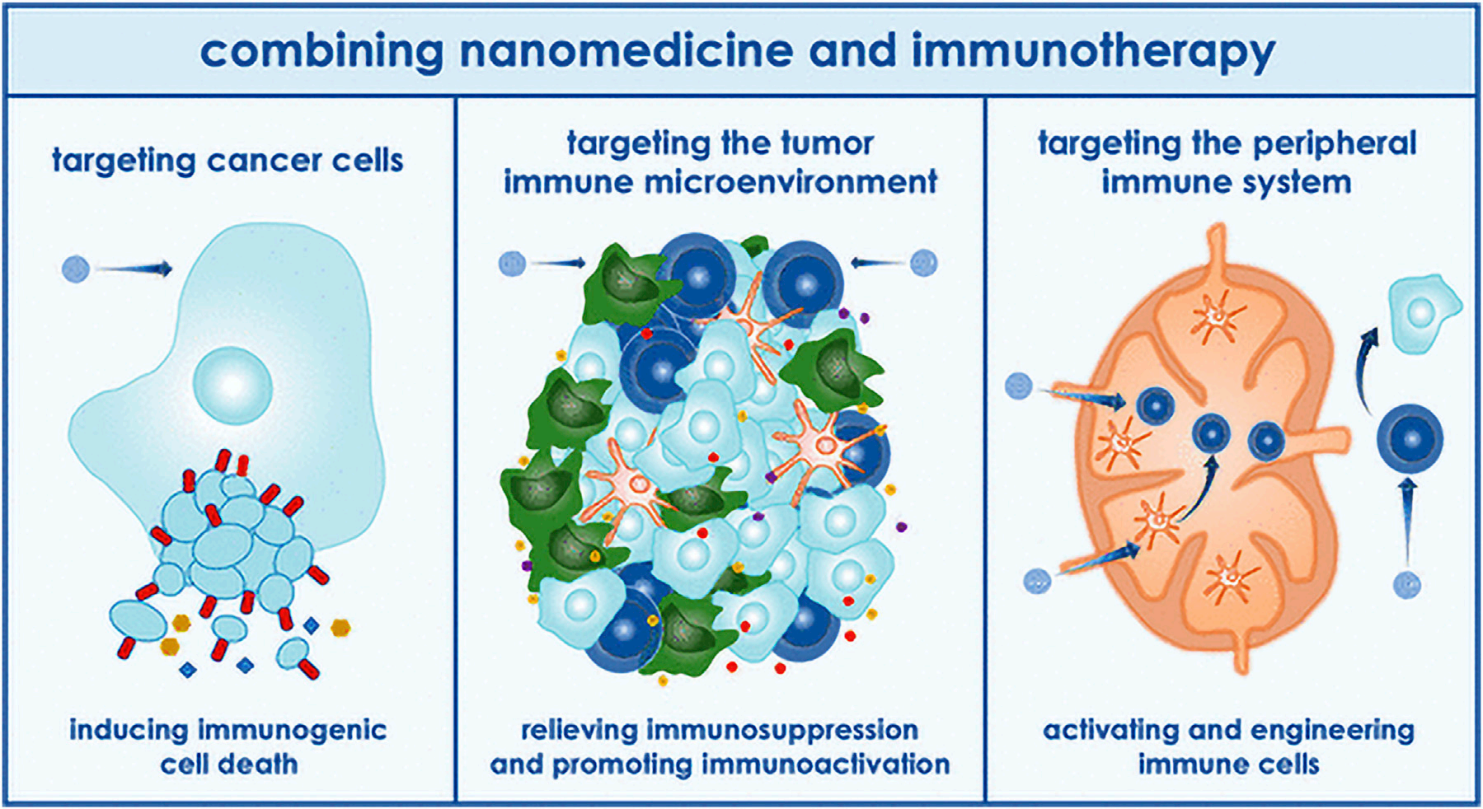
Application of nanoparticles in immunotherapy (Shi and Lammers, 2019).

**FIGURE 8:**
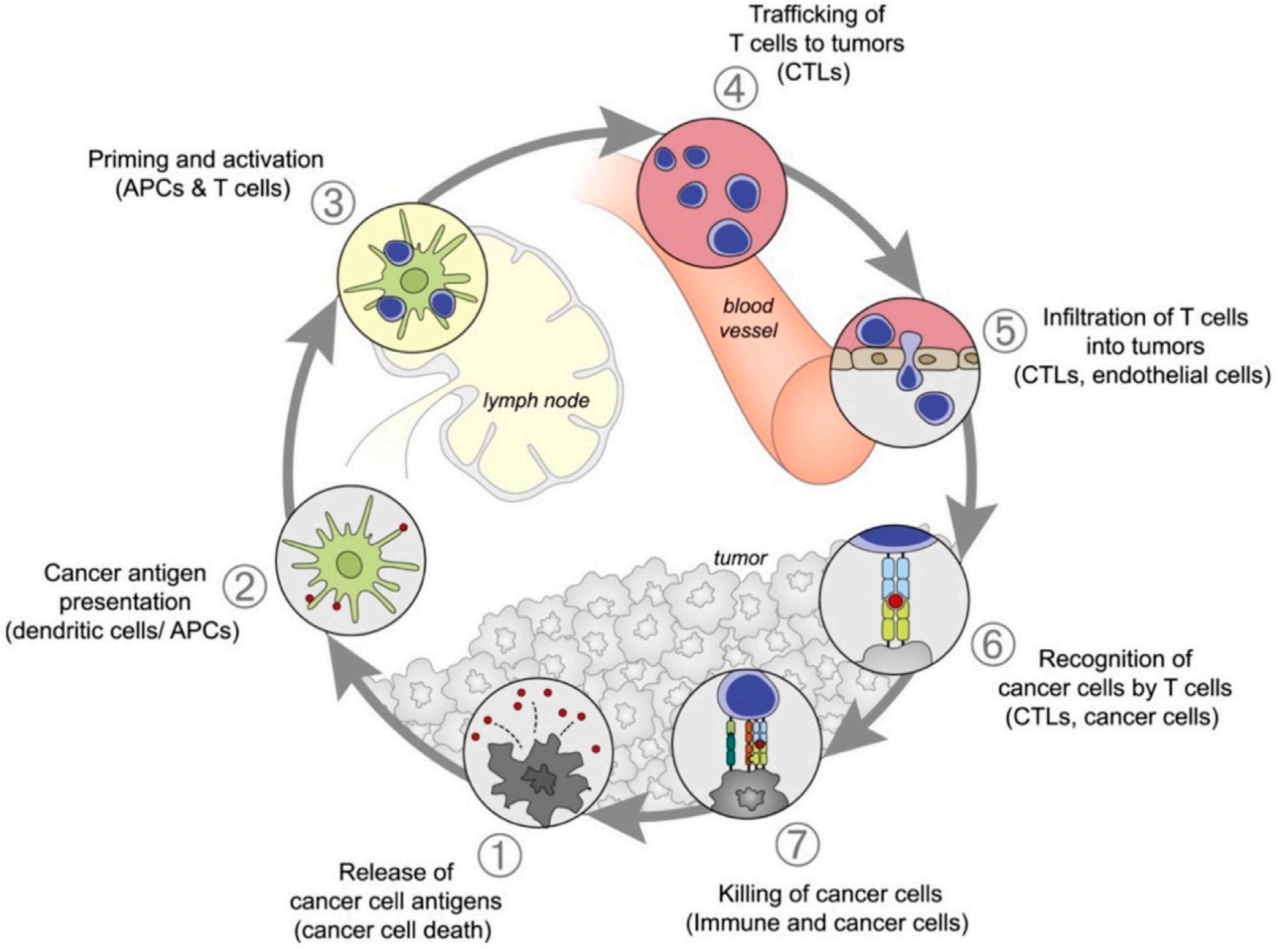
Anti-cancer immune reaction process (Shi and Lammers, 2019).

**FIGURE 9:**
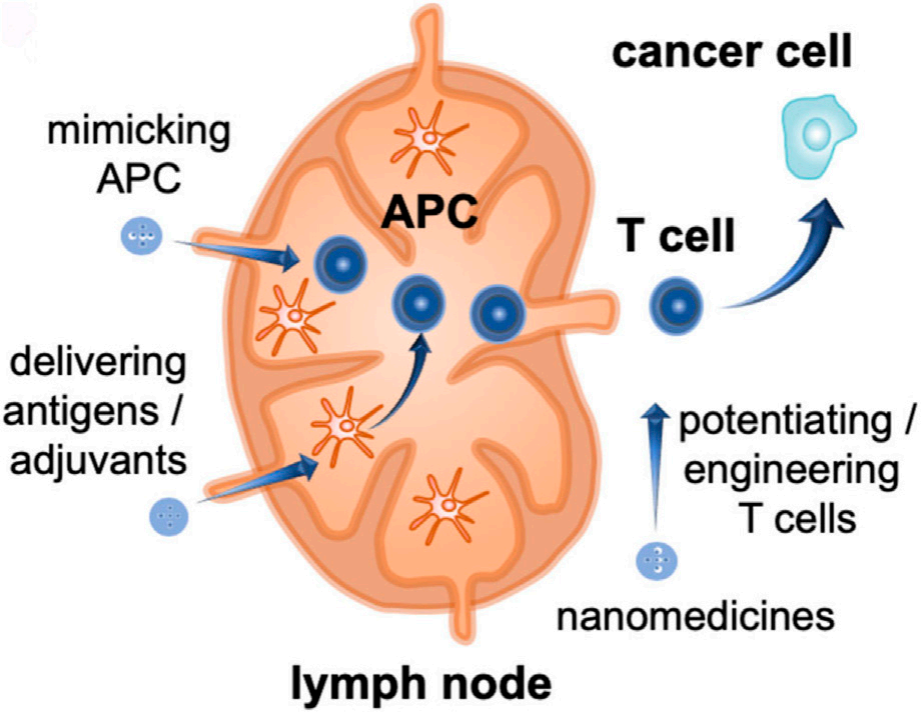
Nanodrugs act on cytotoxic T cells (Shi and Lammers, 2019).

Multiple myeloma (MM) accounts for approximately 10% of all hematological malignancies. Although MM is still considered a single disease, it is a collection of several malignant plasma cell tumors with different cytogenetics. Almost all MM patients will eventually relapse. The choice of the treatment plan is very complex when relapse occurs. It is affected by many factors, including recurrent t-type insertion, previous treatment adaptability, recurrence severity, and performance status (Rajkumar, 2019). Immunotherapy is currently the most commonly used treatment scheme that improves the survival rate of patients with newly diagnosed and relapsed/refractory multiple myeloma (Verkleij et al., 2020). Nanomedicine has excellent potential to enhance the efficacy of cancer immunotherapy. Nanoimmunotherapy can be realized by three different methods, as shown in Figure 7, for 1) targeting cancer cells, 2) targeting the tumor immune microenvironment, and 3) targeting the peripheral immune system (Shi and Lammers, 2019). The anti-cancer immune response begins with the release of cancer cell antigens, which are absorbed, processed, and presented by antigen presenting cells (APC) to the secondary lymphoid organs of naïve T cells, including lymph nodes and spleen (Figure 8). Subsequently, cytotoxic T lymphocytes (CTL) are produced, which migrate and infiltrate tumors and metastatic tumors. CTL can identify and kill cancer cells in tumors and metastatic tumors. By modulating antigen presentation or stimulating APC, nano drugs may enhance the production of cytotoxic T cells in lymph nodes and other peripheral immune organs. In addition, nano drugs can enhance or design cytotoxic T cells to improve the efficiency of cancer cell elimination. The combination of nanotechnology and surface-enhanced Raman probe can significantly facilitate the design of cytotoxic T cells (Figure 9).

Nucleic acid biomarkers for cancer immunotherapy have been expanded to include SERS-active nanomaterials. SERS-active nanoparticles have been created for immunotherapy layering and SERS immunomarker sensing (Dey et al., 2020). SERS uses plasma-enhanced excitation and scattering to obtain high sensitivity while inheriting Raman spectroscopy’s wealth of chemical fingerprint information. For instance, most Raman peaks may be detected readily in an aqueous environment and have small widths suited for numerous studies (Zong et al., 2018). Raman spectroscopy can diagnose multiple myeloma by differentiating between symptomatic and asymptomatic exosomes, identifying spectral differences between active and inactive B lymphocytes, illuminating the mechanism by which B cells control MM, and providing indicators for clinical diagnosis. Nano immunotherapy and SERS nanoprobe are combined to enable targeted therapy in the treatment of MM, greatly enhancing the therapeutic impact. SERS liquid biopsy tracks the changing course of the disease, offering recommendations for damage-free treatment and maximizing drug dosage adjustment. Using multivariate analysis and the SERS spectra of unlabeled blood, Chen et al. examined the non-invasive differentiation of multiple myeloma (MM). 53 MM patients and 44 healthy controls had their high-quality SERS spectra collected using colloidal silver nanoparticles (AgNPs) as a SERS substrate. The differences between the SERS spectra of MM patients and healthy controls indicated that the relative content of biomolecules in their serum was different. Multivariate analysis was used to construct a discriminant model for MM, and it was found that SERS combined with multivariate analysis was a fast, non-invasive, cost-effective technology for identifying MM (Chen et al., 2022). Russo et al. demonstrated the ability of Raman spectroscopy to distinguish exosomes in the conversion from monoclonal gammaglobulin to asymptomatic MM (aMM) and symptomatic MM (sMM), thus providing useful clinical indications for patient diagnosis. The nanostructure-based SERS shows good sensitivity potential (Russo et al., 2020).

Although some patients have experienced a long remission or recovered after functional treatment, others still have early recurrence or receive ineffective treatment. To improve the treatment and prognosis results, it is necessary to comprehensively analyze the information on molecular abnormalities that lead to differences in these results and use biomarker-driven personalized treatment methods. To achieve this goal, biomarkers must be measured robust and repeatable manner. The progress of biomarker monitoring technology helps identify and validate treatment-related biomarkers of myeloma, such as biomarkers that can predict the outcome of patients’ disease according to differences (prognostic biomarkers) or biomarkers that can target the patient’s cell subsets according to a specific molecular pathology (predictive biomarkers) (Pawlyn and Davies, 2019). Raman scattering can be applied to the surface of nanoparticles to create SERS nano threads for indirect detection of biomolecules, which can be used for labeling (indirectly) detection. SERS-active nanoparticles can also achieve intrinsic scattering from biomolecules of interest adsorbed on the surface of nanoparticles. Weak Raman signals can be further improved by adding rough metal surfaces. When molecules are adsorbed on or close to the increased metal surface, this process takes place. The interaction of light and plasmons that have been excited on the metal surface leads to SERS enhancement. The surface of the nanoparticles can be added with Raman detectors to create (indirect) detection of SERS nano label labeling that can be used to indirectly detect biomolecules and obtain intrinsic scattering from the target biomolecules adsorbed on the nanoparticles for use without labeling (direct) capacity.

SERS nanotags can also achieve targeting functions through biomolecular detection and can simultaneously detect various biomarkers. This indicates that the technology has significant advantages in cancer detection and diagnosis with the simultaneous detection of multiple biomarkers (Sloan-Dennison et al., 2021). The development of MM may be significantly influenced by B cells (particularly B-cell subsets), as plasma cells are derived from B cells. The growth of malignant plasma cells may be inhibited directly or indirectly by an increased B-cell population. Another cause can be an increase in B cells or healthy plasma cells due to the decline in malignant plasma cells. The putative method by which B cells control the growth of MM cells needs further investigation (Liu and Rong, 2021). Morrish et al. linked changes in chemical and structural features with biological outcomes using immunological B lymphocyte development as a model, confocal Raman imaging in conjunction with microfluidic devices, and pertinent transcriptomics to analyze chromatin and transcriptional alterations. Multivariate analysis was performed to differentiate the different cell components in each cell. Then, utilizing high-throughput sequencing technology for sequencing analysis, a typical RNA-seq analysis is used to reflect the expression level of mRNA, smallRNA, non-codingRNA, *etc.* They identified the spectrum distinctions between dormant and active B cells and evaluated their relationship to recognized intracellular biological changes.

**FIGURE 10:**
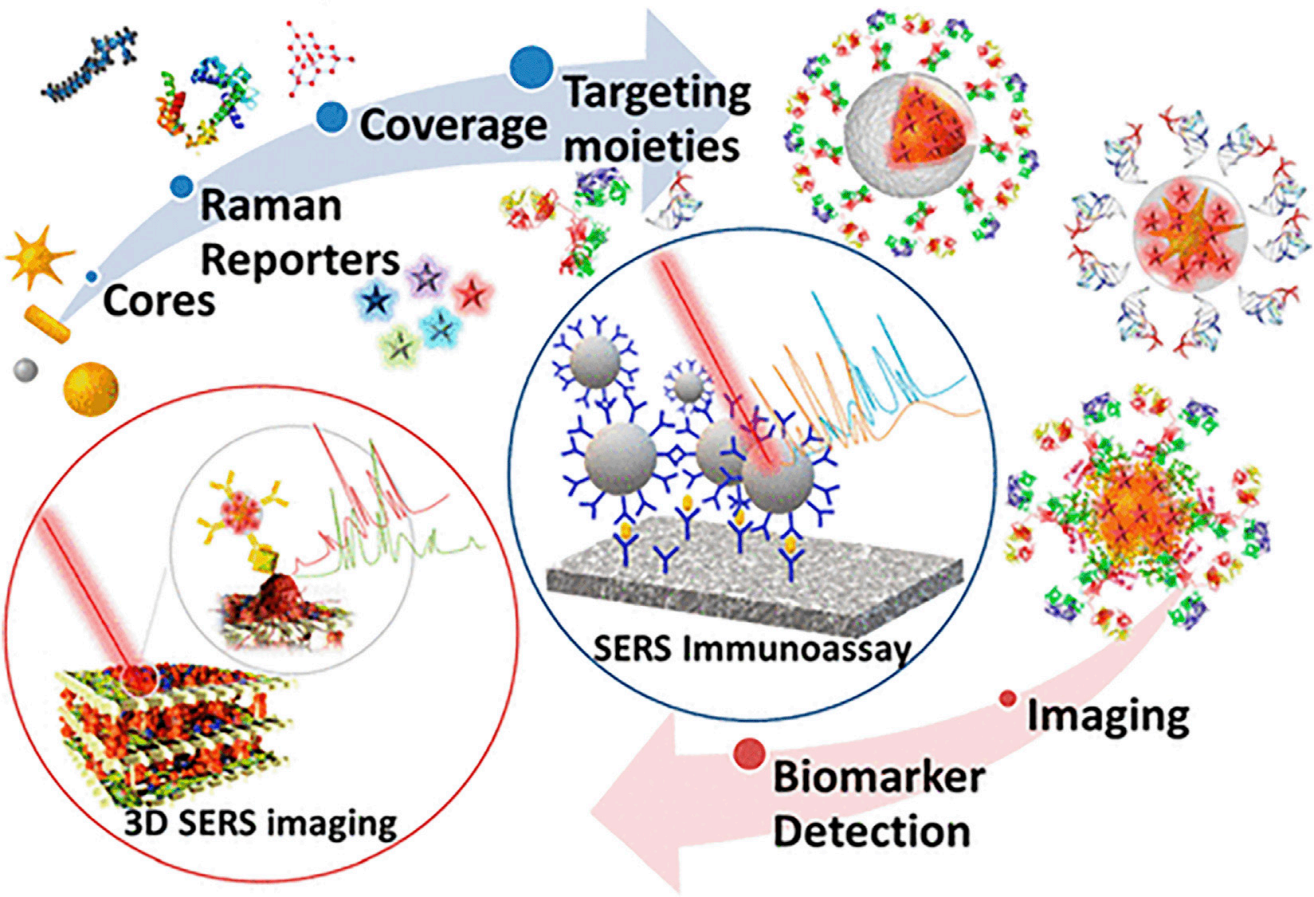
Application of surface-enhanced Raman spectroscopy in biomarker detection (Lenzi et al., 2019).

The limitations of conventional molecular biology technology may be supplemented by Raman spectrum analysis, which offers a potent tool for examining gene mutations. It also provides a technique to trace the dynamics of the biochemical composition of a single cell (Morrish et al., 2021). Precision medicine for MM still faces several difficulties, primarily because no one driver mutation causes MM, making it unlikely that all patients would benefit from the development of selectively targeted treatment. On the other hand, using liquid biopsy in diagnosis to gauge the complexity of the illness may provide less invasive approaches for guiding therapy and monitoring the dynamic nature of the disease process. This approach offers many benefits, including better treatment guidance, a shift toward more individualized treatment options, a reduction in unintended side effects, and an improved approach to medication dose management (Ferreira et al., 2020). SERS technology is used in applying liquid biopsy technology because of its non-destructive nature and high sensitivity. It is used to identify antibodies of selected biomarkers selectively. Compared with ELISA, it has increased multiple detection capabilities. (Dell'Olio et al., 2021b). Figure 10 shows SERS’s scatter labeling and biomarker detection for three-dimensional biological imaging. The ultrasensitive and multiple detections of cancer-related proteins based on SERS technology will be recognized as the theme of research work in the next decade.

## Conclusion and outlook

A novel supplementary tool for illness diagnostics is Raman spectroscopy. However, there are still certain drawbacks, such as substances’ unsatisfactory spontaneous Raman spectrum signal. They have a limited application range, low sensitivity, and are susceptible to fluorescence interference. Resonance Raman scattering was developed to identify cytosine methylation without labeling, amplification, library construction, or other operations. It does this by enhancing the material’s Raman spectrum signal, which significantly improves the sensitivity limitation of traditional Raman spectroscopy. The resolution of Raman spectroscopy is precise in creating intermolecular chemical bonds, and the strong coupling state of atoms may also be identified; Coherent Raman imaging is applied to live cell imaging to determine intracellular lipid, protein, nucleic acid, and water levels with great sensitivity.

Raman spectroscopy provides clear benefits over conventional hematological tumor diagnosis techniques. Its main advantages are that it can produce a Raman fingerprint without causing any damage, is specific, and has high sensitivity. Moreover, it does not require any sample preparation or fluorescent or isotope labeling during the detection process, which perfectly satisfies the demands of quick and real-time clinical detection. Raman spectroscopy may also provide precise discrimination and micro-detection with minor sample damage, which is very important for analyzing the course of a disease and its prognosis after therapy. Of course, combining Raman spectroscopy technology with conventional biochemical evaluation techniques may be optimal for its clinical application. Raman’s distinctive spectral peaks provide accurate identification. As additional tools for laboratory diagnosis, biochemical detection markers are used. The two, taken together, will significantly increase clinicians’ ability to make accurate diagnoses. For instance, exactly how most blood system cancers form and occur is still unclear. Low clinical diagnosis efficiency is caused by biochemical markers of the blood system that can be impacted by other primary diseases and have complex changes that frequently require medical expertise to interpret and appraise. Hematological tumors can be identified and treated, and the prognosis can be determined using Raman scattering and its registration techniques. Raman spectroscopy is increasingly being used in healthcare settings.
